# Neuronal and Astroglial Localization of Glucocorticoid Receptor GRα in Adult Zebrafish Brain (*Danio rerio*)

**DOI:** 10.3390/brainsci13060861

**Published:** 2023-05-26

**Authors:** Evangelos Natsaridis, Panagiotis Perdikaris, Stefanos Fokos, Catherine R. Dermon

**Affiliations:** Laboratory of Human and Animal Physiology, Department of Biology, University of Patras, Rion, 26504 Patras, Greece; natsaridis.e@gmail.com (E.N.); perdikarisp@gmail.com (P.P.); stefanos_fokos@yahoo.gr (S.F.)

**Keywords:** glucocorticoid receptor alpha isoforms, limbic forebrain areas, locus coeruleus, immunohistochemistry, Western blot, catecholaminergic, radial glia

## Abstract

Glucocorticoid receptor α (GRα), a ligand-regulated transcription factor, mainly activated by cortisol in humans and fish, mediates neural allostatic and homeostatic functions induced by different types of acute and chronic stress, and systemic inflammation. Zebrafish GRα is suggested to have multiple transcriptional effects essential for normal development and survival, similarly to mammals. While sequence alignments of human, monkey, rat, and mouse GRs have shown many GRα isoforms, we questioned the protein expression profile of GRα in the adult zebrafish (*Danio rerio*) brain using an alternative model for stress-related neuropsychiatric research, by means of Western blot, immunohistochemistry and double immunofluorescence. Our results identified four main GRα-like immunoreactive bands (95 kDa, 60 kDa, 45 kDa and 35 kDa), with the 95 kDa protein showing highest expression in forebrain compared to midbrain and hindbrain. GRα showed a wide distribution throughout the antero-posterior zebrafish brain axis, with the most prominent labeling within the telencephalon, preoptic, hypothalamus, midbrain, brain stem, central grey, locus coeruleus and cerebellum. Double immunofluorescence revealed that GRα is coexpressed in TH+, β_2_-AR+ and vGLUT+ neurons, suggesting the potential of GRα influences on adrenergic and glutamatergic transmission. Moreover, GRα was co-localized in midline astroglial cells (GFAP+) within the telencephalon, hypothalamus and hindbrain. Interestingly, GRα expression was evident in the brain regions involved in adaptive stress responses, social behavior, and sensory and motor integration, supporting the evolutionarily conserved features of glucocorticoid receptors in the zebrafish brain.

## 1. Introduction

Glucocorticoids are essential for life, mediating homeostatic/allostatic adaptations in response to stress [1], and play an important role for many physiological processes, including immune function, reproduction, cardiovascular and neural function. Mainly due to their strong anti-inflammatory functions, glucocorticoids are widely applied to treat acute or chronic inflammation [2]; however, glucocorticoids have multiple effects in the brain, and their chronic administration is known to influence adaptive stress responses and may induce neuropsychiatric conditions, such as affective disorders, including depression and anxiety. Their diverse physiological actions are mediated via glucocorticoid receptor (GR), a ligand-responsive nuclear receptor [3], by inducing or repressing the transcription of several target genes (up to 10–20% of human genome) [4,5,6]. GRs are present in all vertebrates, supporting an evolutionarily well-conserved stress response mechanism. In teleost fish, as is in humans, cortisol (in contrast to corticosterone in rodents) is the major glucocorticoid hormone, which increases in response to stress, regulated by the hypothalamus-pituitary-interrenal (HPI) axis, suggested as equivalent to the mammalian hypothalamus-pituitary-adrenal (HPA) axis, by a negative feedback mechanism [7].

GR modular protein has three major domains: an N-terminal transactivation domain, involved in basal transcription and post-translational modifications; a central DNA-binding region, a highly conserved region throughout vertebrates that binds to the glucocorticoid responsive elements (GREs); and a C-terminal ligand binding domain, a relatively conserved region, that forms a hydrophobic pocket for binding glucocorticoids in a ligand-dependent manner [8,9]. Several studies have discovered a range of receptor subtypes arising from alternative processing of a single GR gene, with different expression pattern, gene regulatory and functional profiles. Post-translational modification expands GR diverse signaling [10] and, in turn, cellular responses to glucocorticoids [11,12]. In mammals, glucocorticoid binding to GRs in the cytoplasm [13,14] is known to result in the rapid translocation of GRs into the nucleus where they bind to GREs, regulating the transcription of target genes [15,16] or acting by non-genomic mechanisms on cell signaling processes [17,18]. Polymorphisms in the GR gene altering the amino acid sequence of the encoded receptor affect GR function as a transcriptional activator or repressor [19,20]. In humans, the GR gene consists of nine exons that their alternative splicing generates two receptor isoforms, GRα and GRβ. GRα is considered the main GR mediating glucocorticoid actions while the alternative translation of GRα mRNA transcript additionally produces several GR proteins. Particularly, eight GRα isoforms, with GRα-A isoform known as the full-length receptor, conserved among mammals having progressively shorter N-terminal domains, are derived from exon 2 of the GR gene [10,12,19].

Zebrafish (*Danio rerio*) have been established as an important model organism, complementing the widely used rodent models for neuropsychiatric research, and stress-related diseases, including depression [21]. The zebrafish genome is suggested to contain a single *gr* gene [22], while other teleost (salmonids and percomorphs) were shown to have two different GR genes, gr1 and gr2 [23,24], with high sequence similarity, particularly in the gene section coding the C-terminal of the receptor protein [25]. Interestingly, in a zebrafish grs357 mutant, chronic disruption of GR activity induced CRH, ACTH and cortisol elevation, and an exaggerated behavioral stress response, suggesting that dysfunction of GR-mediated transcriptional regulation can induce an affective disorder [26]. 

Although the distribution of the glucocorticoid receptor in the adult brain of primates [27,28], rodents [29,30] and Salmonidae teleost [31,32] has been reported, the neuroanatomical distribution and cellular localization of GRα in the zebrafish brain has not yet been described. For this, the present study questioned the glucocorticoid receptor isoforms as well as the regional cerebral distribution and cellular localization of GRα in the adult zebrafish (*Danio rerio*), by means of Western blot, immunohistochemistry and double immunofluorescence. Taking into account that zebrafish is an alternative useful model organism for studying neuropsychiatric disorders and the key-role of GRs in adaptive and maladaptive brain functions, the present study would add significant new knowledge in understanding the GR-mediated physiological and pathophysiological mechanisms. 

## 2. Materials and Methods

### 2.1. Animals 

Adult (n = 12, seven to twelve months old) wildtype zebrafish (Cyprinidae, *Danio rerio*) of both sexes were kept in aged water at 28 °C, under a 12:12 h light/dark cycle. All experimental procedures followed the European Communities council directive 86/609/EEC for the care and use of laboratory animals and were approved by the ethics committee of University of Patras and by the Veterinary Administration of the Prefecture of Achaia, Greece (approval no. 110156/411).

### 2.2. Western Immunoblotting

**Figure 1:**
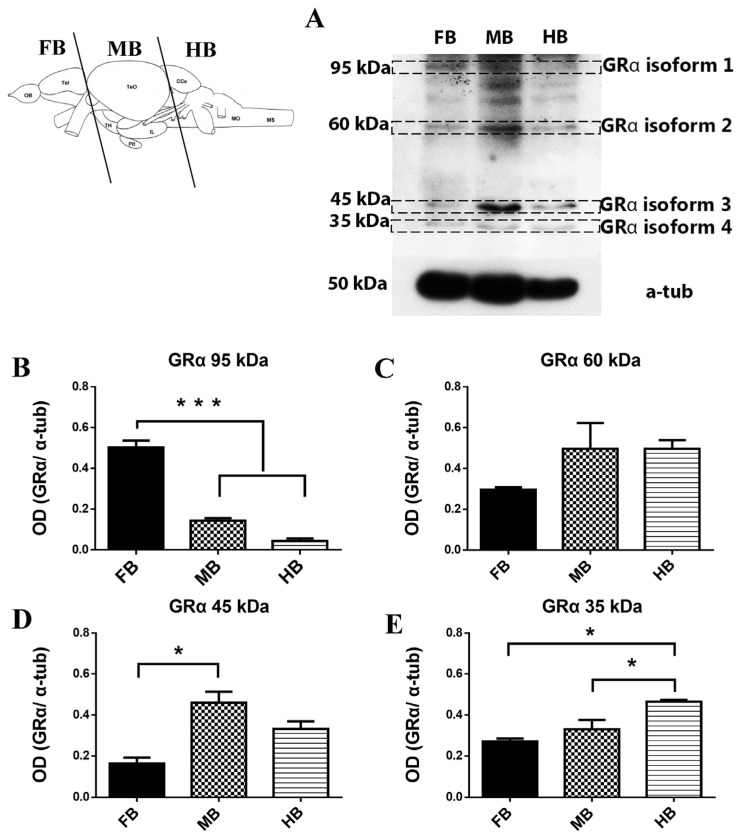
Western immunoblotting showing the quantitative expression of glucocorticoid receptor (GRα) isoforms in the adult zebrafish brain. In the top left, the coronal levels used for separation and isolation of forebrain (FB), midbrain (MB) and hindbrain (HB) are shown. (**A**) Western blot images of the different GRα-like isoforms in the forebrain, midbrain and hindbrain, considered as isoforms 1 to 4. (**B**–**E**) Quantitative optical density (OD) measurements of the expression levels of GRα-like isoforms in the zebrafish forebrain, midbrain and hindbrain. Values represent an average +/− standard error in absolute values (N = 4). Symbol (***) indicates statistically significant differences in GRα expression in forebrain compared to midbrain and hindbrain, respectively in A (*** *p* < 0.001), Symbol (*) indicates statistically significant differences in forebrain compared to the hindbrain in (**D**) (* *p* < 0.05). Symbol (*) also indicates statistically significant differences in hindbrain compared to GRα expression in the forebrain and midbrain, respectively, * *p* < 0.05, in (**E**).

In order to identify the several GRα isoforms, zebrafish (*Danio rerio*, n = 6) brains were separated at the levels shown in Figure 1, in three parts (forebrain, midbrain and hindbrain) and homogenized with a Teflon–glass homogenizer in cold RIPA lysis buffer containing 50 mM Tris-HCL, pH 8.0; 150 mM NaCl; 1% NP-40; 0.5% sodium deoxycholate; 0.1% SDS; and protease inhibitor cocktail (Roche Life Science, Penzberg, Germany). Tissue homogenates were incubated on ice for 30 min (vortex every 10 min), centrifuging at 3300 rpm for 5 min at 4 °C to remove cell debris. Supernatants were collected, and proteins were electrophoretically separated (30 micrograms protein) in a 9% SDS-polyacrylamide gel following their concentration determination using BCA protein assay (Thermo Fisher Scientific, Rockford, IL, USA). The separated proteins were transferred to polyvinylidene fluoride (PVDF) membranes (Merck Millipore, Temecula, CA, USA) at 350 mA for 1.5 h at 4 °C. Following 75 min of blocking (5% non-fat dried milk) in tris-buffered saline (TBS)-Tween (0.05% Tween-20 in 0.01 M TBS), the proteins were incubated with the GRα rabbit polyclonal antibody (sc-1002, Santa Cruz Biotechnology, Dallas, TX, USA; 1:100 for 30 μg in TBS-Tween 2% nonfat dried milk) at 4 °C for 15–18 h. Then, after 3washes in TBS-Tween, 10 min each, the membranes were incubated with secondary anti-rabbit IgG antibodies (AP132P, Millipore; 1:20,000 in TBS-Tween 2% nonfat dried milk) for 1.5 h at room temperature and washed 3 times in TBS-Tween, 10 min each and 2 times in TBS, 5 min each. For labeled protein band visualization the Immobilon Western Chemiluminescent HRP Substrate (Merck Millipore) was applied, according to the manufacturer’s instructions, and α-tubulin was used as a loading control (Τ5168, SIGMA, St. Louis, MO, USA; 1:6000 for 30 μg in TBS-Tween 2% nonfat dried milk).

### 2.3. Immunohistochemistry and Double Immunofluorescence

Zebrafish (n = 6) were intracardially perfused with 4% paraformaldehyde (PFA, Sigma-Aldrich, St. Louis, MO, USA) under deep anesthesia (0.1% tricaine methane sulfonate, MS-222). Their brains were carefully removed, post-fixed in 4% PFA in PBS for 2 h, cryoprotected overnight in 20% sucrose in 0.1 M phosphate buffer (PB; pH 7.4) at 4 °C. Following freezing in dry-ice-cooled 2-methyl butane (Sigma-Aldrich) at approximately −35 °C, the brains were stored at −80 °C until use. Coronal sections, 20 μm thick, prepared using a Leica cryostat, were collected on gelatin-coated slides and were immediately processed for immunohistochemistry or double immunofluorescence.

Single-labeling experiments were performed for the determination of the GRα distribution pattern. Briefly, following a 15 min wash in 0.01 M PBS, pH 7.4 (3× washes, 5 min each), sections were incubated with 3% H_2_O_2_ (Sigma-Aldrich) in PBS, 10 min, at room temperature to inhibit endogenous peroxidase activity. Non-specific protein binding sites were blocked with 1% normal horse serum (NHS), with 5% bovine serum albumin (Sigma-Aldrich) and with 0.5% Triton X-100 in PBS for 40 min. Sections were then incubated for 15–18 h at 4 °C in a moist chamber with rabbit anti-GRα (Santa Cruz Biotechnology, 1:100 in PBS with 0.5% Triton X-100, 1% NHS and 1% BSA). Following 3 rinses, 5 min each, in PBS, sections were incubated with a biotinylated anti-rabbit antibody (Vector, Tokyo, Japan, 1:200 in PBS) for 2.5 h at room temperature, washed 3 times in PBS with 0.5% Triton X-100 and incubated in the dark with Vectastain Elite ABC reagent (Vector Laboratories; 1:100A and 1:100B) in PBS with 0.5% Triton X-100n, 1 h at room temperature, washed in PBS, followed by 3,3′-diaminobenzidine (DAB; Vector) reaction for visualization and then dehydrated and cleared with xylene and cover slipped with Entellan. 

To determine the phenotype of GRα immunolabeled cells, sections were incubated with a solution of polyclonal anti-GRα (1:100 in PBS with 0.5% Triton X-100) with monoclonal anti-tyrosine hydroxylase (anti-TH, 1:1000 in PBS with 0.5% Triton X-100) for staining dopaminergic neurons; with monoclonal anti-glial fibrillary acidic protein antibody (anti-GFAP, a glial cell marker, 1:1000 in PBS with 0.5% Triton X-100) for labeling glial cells; or with anti-vesicular glutamate transporter (anti-vGLUT) monoclonal antibody (1:1000 in PBS with 0.5% Triton X-100) for labeling glutamatergic neurons, or with monoclonal β_2_-adrenergic receptor (β_2_-AR), for 15–18 h at 4 °C. Details of the primary antibodies used are shown in Supplementary Table S1. Anti-rabbit Alexa fluor 488, anti-goat Alexa fluor 568, or anti-mouse Alexa fluor 555 (Molecular Probes, Leiden, The Netherlands; diluted 1:400 in 0.5% Triton X-100 in PBS) were used as appropriate cocktail for secondary antibodies for 2.5 h in the dark at room temperature. Then, following PBS rinsing, sections were cover slipped with fluorescent hard medium (Vector, H-1400). 

Control experiments with the omission of each primary antibody, and/or application of secondary antisera mismatched for species were performed in adjacent sections and resulted in no staining in all cases.

### 2.4. Antibody Characterization

The P-20 anti-GRα primary antibody (sc-1002X, Santa Cruz Biotechnology), targets a region within amino acids 720–770 of the GRα protein, shown to recognize GRα isoforms by cloning and in vitro transfection [33].This anti-GRα antibody binds in the C terminal area of the human glycocorticoid receptor alpha and has been successfully used in animal models, including zebrafish [34,35,36,37,38], rats [39,40], as well as in human tissue [5,33].The antibody used in the present study has been determined previously to identify an immunoreactive protein of 95 kDa suggested to correspond to the zebrafish GRα [34]. 

To determine the specificity of the P-20 sc1002 GRα antibody under our running conditions, Western blot experiments with preincubation with an excess of the blocking peptide (C(755) E I I T N Q I P K Y S N G N I K K (771)) were conducted. In addition, Western immunoblot experiments were conducted to compare the migration of the immunoreacting proteins in mammalian and teleostean brains (Supplementary Figure S1).

Antibodies used for identifying neuronal and glial cell populations expressing glucocorticoid receptors have been previously characterized, and the cellular morphology and the distribution staining pattern observed in the present study was similar to those previously reported in the teleost nervous system. Specifically, the monoclonal anti-tyrosine hydroxylase (anti-TH), IgG1kappa, clone LNC1, at approximately 59–61 kDa was used for staining dopaminergic neurons, and the present data agree with previously described pattern in adult zebrafish brain [41,42]. Monoclonal anti-GFAP antibody (Sigma, IgG1 isotype, clone G-A-5) recognize a band at approximately 51 kDa corresponding to GFAP, a class-III intermediate filament found in astrocytes and radial glia, and some types of ependyma cells in most vertebrates. The GFAP staining in this study is similar to that previously reported for labeling glial cells in adult zebrafish brain [41,43]. The vesicular glutamate transporter 2 antibody (anti-vGLUT2), a recombinant full-length rat vesicular glutamate transporter 2 (vGLUT2), clone 8G9.2T (manufacturer’s datasheet), has been previously used for labeling vesicle glutamate transporters [44,45] with similar labeling pattern. In addition, the β_2_-AR antibody has been previously used to study the anxiety-like behavior in MK-801 adult zebrafish model [46]. 

### 2.5. Brain Microscopy, Photomicrograph Processing

The identification of brain regions was based on the zebrafish brain atlas of Wullimann et al., 1996 [47]. A CFW-1600 digital camera (Color CCD, depth 10 bit, Scion, Chicago, IL, USA) adjusted on an optical and fluorescent microscope (Nikon, Singapore, Eclipse E800) connected to a PC was used for image processing, capturing and digitizing microscopic images. NIH ImageJ software, Version 1.53m, (National Institutes of Health, Bethesda, MD, USA) [48] was applied to generate stacks of optically sliced images and to identify double-labeled cells. Adobe Photoshop CS3 (Adobe Systems, San Jose, CA, USA) was used to prepare Figures, and Graph pad Prism 5 was used to prepare graphs.

### 2.6. Westen Blot Quantification and Statistical Analysis

Western blot quantification was based on labeled bands optical density (OD) measurements using NIH ImageJ software (National Institutes of Health). The protein signal intensities were normalized against the corresponding α-tubulin signal. Values are expressed as mean ± SEM. 

The statistical program SPSS was applied, and analysis of GRα protein expression levels in forebrain, midbrain and hindbrain was performed using one-way ANOVA. A probability level of 5% (*p* < 0.05) was considered statistically significant.

## 3. Results

### 3.1. Western Blotting of Glucocorticoid-like Receptors (GRα) in the Adult Zebrafish Brain 

Western immunoblotting experiments revealed the expression of different glucocorticoid-like receptor immunoreactive proteins (GRα) within the forebrain, midbrain and hindbrain (Figure 1). Specifically, in the present study, zebrafish brain highlighted GRα glucocorticoid receptor immunoreactive bands located at 95, 60, 45 and 35 kDa, which appear to be expressed in different concentrations in the forebrain, midbrain or hindbrain. Initial experiments showed that GRα protein expression levels did not differ among male and female brain and thereafter were grouped together. One-way ANOVA statistical analysis showed that the expression levels of GRα-immunoreactive protein at 95 kDa (considered isoform 1) exhibited significantly higher expression in the forebrain compared to the midbrain and hindbrain (F (2,9) = 117.092, *p* = 0.000; Figure 1B). In contrast, glucocorticoid GRα immunoreactivity at 45 kDa (considered as isoform 3) was higher in the midbrain compared to the forebrain (F (2,9) =12.869, *p* = 0.002; Figure 1D). The 35 kDa immunoreactive protein (considered as isoform 4) was significantly higher in the hindbrain compared to the forebrain and midbrain (F (2,9) =12.308 *p* = 0.003; Figure 1E). Expression levels of 60 kDa GRα immunoreactivity (considered as isoform 2) showed similar pattern in the three parts of the brain.

The specificity of the immunoreactivity of the protein bands in adult zebrafish brain was established by a comparison of rat and zebrafish brain immunoreactive bands (Supplementary Figure S1A) and by preincubation with excess of the relative peptide used to raise the antibody (Supplementary Figure S1B). The latter resulted in no specific staining in either rat or zebrafish brain.

### 3.2. Cellular Distribution of the Glucocorticoid Receptor GRα in Zebrafish Brain 

The immunohistochemical labeling of GRα glucocorticoid-like receptors showed a wide GRα immunoreactivity (GRα-ir), distributed similarly in males and females throughout the anteroposterior axis of zebrafish brains. Groups of GRα-immunoreactive cells were found in the telencephalon, diencephalon, mesencephalon, and rhombencephalon, and in most cases, the staining of large GRα positive cells was localized in the cytoplasm, while a percentage of medium- and small-sized cells exhibited both cytoplasmic and nuclear staining. 

#### 3.2.1. Telencephalon

**Figure 2:**
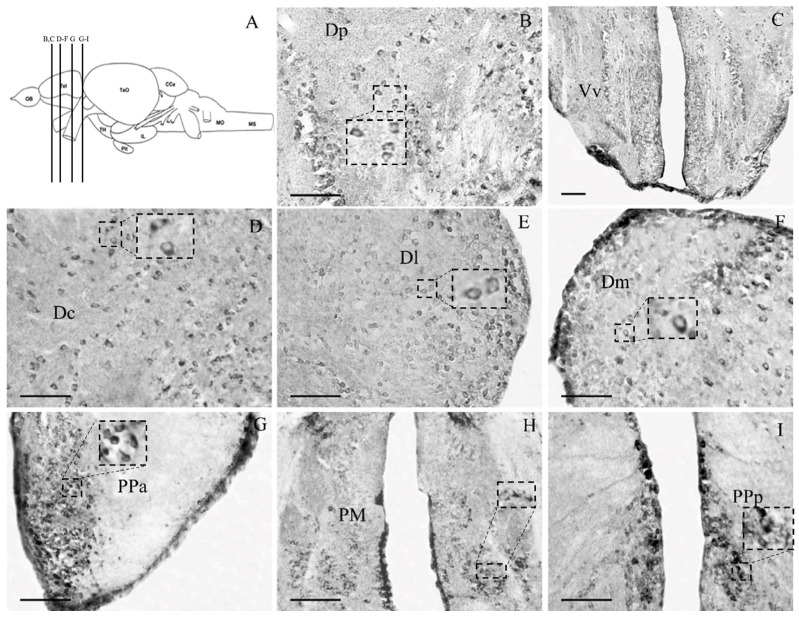
Microphotographs of forebrain areas depicting the cellular pattern of GRα-ir at the levels shown in (**A**) from the atlas of Wullimann et al. (1996) (**B**) Posterior zone of the dorsal telencephalic area, Dp; (**C**) ventral nuclei of ventral telencephalic area, Vv; (**D**) central zone, Dc; (**E**) lateral zone, Dl; and (**F**) medial zone, Dm, of the dorsal telencephalic area; (**G**) anterior part of parvocellular preoptic nucleus, PPa; (**H**) magnocellular preoptic nucleus, PM; (**I**) posterior part of parvocellular preoptic nucleus, PPp; and magnocellular preoptic nucleus, PM. Lateral is on the right for (**B**,**D**–**G**). Insert frames of higher magnification show examples of cytoplasmic labeling in cells of dorsal telencephalic and preoptic regions. Scale bar = 0.05 mm.

**Figure 3:**
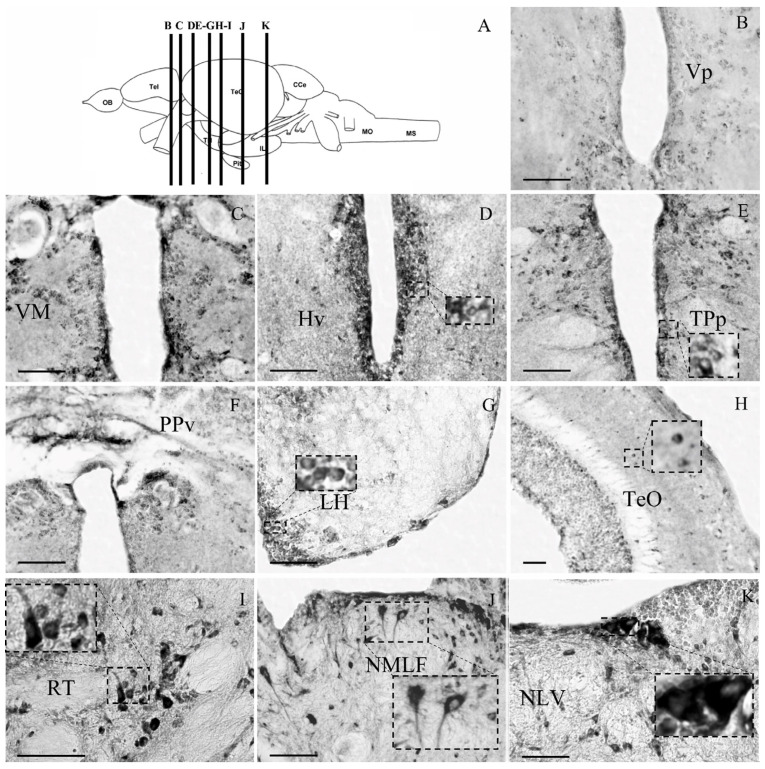
Microphotographs of GRα-ir in zebrafish posterior forebrain and midbrain levels, shown in (**A**), from the atlas of Wullimann (1996) [47]. (**B**) Post-commissural nucleus of ventral telencephalic area, Vp; (**C**) ventromedial thalamic nucleus, VM; (**D**) ventral zone of periventricular hypothalamus, Hv; (**E**) periventricular nucleus of posterior tuberculum, TPp; (**F**) ventral part of periventricular pretectal nucleus, PPv; (**G**) lateral hypothalamic nucleus, LH; (**H**) optic tectum, TeO; (**I**), midbrain nucleus, RT; (**J**) the nucleus of medial longitudinal fascicle, NMLF; and (**K**) nucleus lateralis valvulae, NLV. Lateral is on the right for (**G**–**K**). Insert frames of higher magnification show examples of cytoplasmic labeling. Scale bar = 0.05 mm.

The zebrafish telencephalon includes the dorsal (dorsal telencephalic area) and ventral (ventral telencephalic area) regions, suggested to correspond to the pallium and the subpallium, respectively [49]. Both dorsal and ventral telencephalic areas were found to express moderate levels of glucocorticoid receptors, as shown in Figure 2. Immunohistochemistry mainly demonstrated cytoplasmic GRα expression, which were found in small- and medium-sized immune-reactive cells within the dorsal (Dm), lateral (Dl), central (Dc), and posterior (Dp) zones of the dorsal telencephalic area (Figure 2B,D–F); the latter is considered to be homologue to the olfactory cortex [49]. In addition, GRα glucocorticoid receptor expression was prominent close to the midline dorsal (Vd) and ventral (Vv) nuclei of the ventral telencephalic area (Figure 2C), considered comparable to the mammalian striatum and septum, respectively [49]. In addition, immunoreactivity was observed within small-sized cells in the post-commissural nucleus of ventral telencephalic area (Vp; Figure 3B). 

#### 3.2.2. Diencephalon

A high density of small- and medium-sized GRα-ir cells, with mainly cytoplasmic expression, were found within the anterior parvocellular preoptic nucleus (PPa), in the posterior part of parvocellular preoptic nucleus (PPp), in the magnocellular preoptic nucleus (PM; Figure 2H,I) and the ventromedial thalamic nucleus (VM; Figure 3C). Densely labeled cells were observed in the periventricular zones of the third ventricle, the ventral zone of periventricular hypothalamus (Hv; Figure 3D), the periventricular nucleus of posterior tuberculum (TPp; Figure 3E), the ventral part of periventricular pretectal nucleus (PPv; Figure 3F), and in the medial region of lateral hypothalamic nucleus (LH; Figure 3G).

#### 3.2.3. Mesencephalon

GRα expression was prominent in the midbrain sensory- and motor-related areas involved in visual and multisensory integration processes, such as recognition and position of objects, spatial orientation, and motor coordination [50,51]. Specifically, GRα immunoreactivity was found in small-, medium- and large-sized cells in zebrafish optic tectum (TeO) and midbrain tegmentum. The TeO is a well laminated structure of six layers (stratum marginale, stratum opticum, stratum fibrosum et griseum superficiale (SFGS), stratum griseum centrale (SGC), stratum album centrale (SAC), and stratum periventriculare (SPV or PGZ). GRα-like expression showed a sparse labeling of small positive cells, with higher staining within the tectal layers SFGS and SPV (Figure 3H). Large, densely stained cells were located in the rostral tegmental nucleus (RT; Figure 3I) and the nucleus of medial longitudinal fascicle (NMLF; Figure 3J). GRα expression mainly showed cytoplasmic expression but in cases both cytoplasmic and nuclear staining was observed (RT, Figure 3I), possibly indicating the expression of different receptor isoforms.

#### 3.2.4. Rhombencephalon

**Figure 4:**
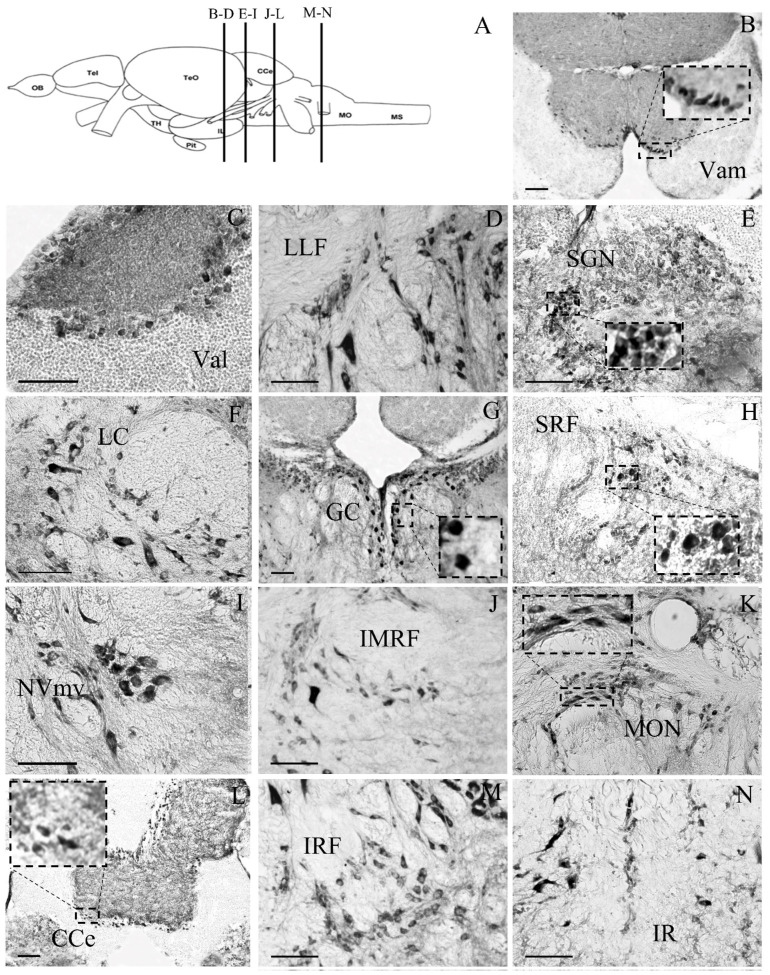
Microphotographs of glucocorticoid receptor expression in zebrafish cerebellum and the rhombencephalic nuclei of zebrafish brains, at the coronal levels shown in (**A**), using the atlas of Wullimann (1996) [47]. (**B**) Medial division of valvular cerebelli, Vam; (**C**) lateral division of valvular cerebelli, Val; (**D**) lateral longitudinal fascicle, LLF; (**E**) secondary gustatory nucleus, SGN; (**F**) locus coeruleus, LC; (**G**) central gray, GC; (**H**) superior reticular formation, SRF; (**I**) trigeminal motor nucleus, ventral part, NVmv; (**J**) intermediate reticular formation, IMRF; (**K**) medial octavolateralis nucleus, MON; (**L**) corpus cerebelli, CCe; (**M**) inferior reticular formation, IRF; and (**N**) inferior raphe, IR. Lateral is on the right for (**C**–**F**,**H**–**K**,**M**,**N**). Insert frames of higher magnification show examples of nuclear (**G**) or cytoplasmic labeling (**A**,**E**,**H**,**I**). Scale bar = 0.05 mm.

GRα expression in the medulla oblongata and cerebellum of zebrafish brains is illustrated in Figure 3K and Figure 4. Specifically, medium and large cells exhibited GRα immunoreactivity in the pre-cerebellar nucleus lateralis valvulae (NLV; Figure 3K). The zebrafish cerebellum, including the valvula cerebelli (Va, Val, Vam), the corpus cerebelli (CCe) and lobus caudalis cerebelli (LCa), showed significant GRα immunoreactivity. Strong labeling within the medial and lateral division of valvula (Vam, Val) and corpus (CCe) cerebelli, was located at the ganglionic cell layer and the granule cell layer (Figure 4B,C,K). Specifically, in the ganglionic cell layer, at the border between the granular and molecular layers, strong Purkinje cell somata immunoreactivity for GRα was found. In addition, large densely labeled cells were determined in the lateral longitudinal fascicle (LLF; Figure 4D). Densely labeled small granule cells were located in the secondary gustatory nucleus (SGN, Figure 4E). Interestingly, the somata and the proximal dendrites of the locus coeruleus (LC) neurons were strongly stained for GRα (Figure 4F). Densely labeled medium-sized cells showing intense nuclear and cytoplasmic labelling were found in the central gray (GC; Figure 4G). Brain stem nuclei, including the oculomotor nucleus (ΝIII), the superior reticular formation (SRF, Figure 4H), the trigeminal motor nucleus ventral part (NVmv; Figure 4I), the intermediate reticular formation (IMRF; Figure 4J), the medial octavolateralis nucleus (MON; Figure 4K), and the inferior reticular formation (IRF; Figure 4M) showed strong immunoreactivity. Small-sized labelled cells were detected in the inferior raphe (IR; Figure 4N). 

### 3.3. Phenotype of Cells Expressing GRα

Interestingly, GRα was found to be colocalized in neurons expressing important neurotransmitter systems, possibly exerting influence on their functions. Specifically, GRα-ir was co-localized in neuronal cells expressing β_2_-adrenergic receptors (β_2_-ARs), catecholaminergic (TH+), and glutamatergic (v-GLUT+, vesicular glutamate transporter 2 positive) markers. GRα glucocorticoid receptor immunoreactivity was also detected in cells expressing GFAP. 

**Figure 5:**
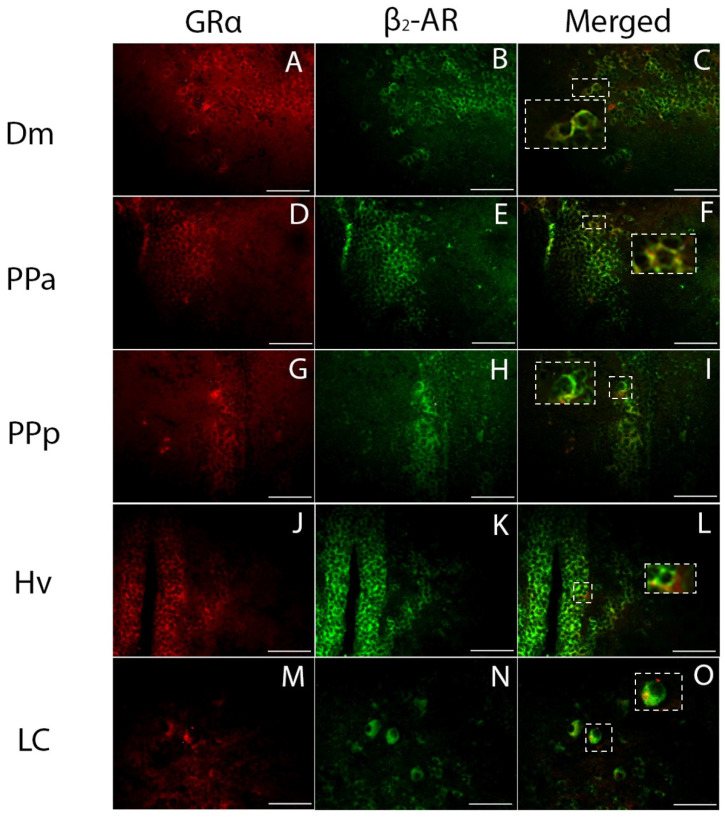
Double immunofluorescence of cells expressing GRα-ir co-localized with the β_2_-ARs in zebrafish brains. Double-labeled cells were found in (**A**–**C**) the medial zone of the dorsal telencephalic area, Dm; (**D**–**F**) the anterior part of the parvocellular preoptic nucleus, PPa; (**G**–**I**) the posterior part of the parvocellular preoptic nucleus, PPp; (**J**–**L**) the ventral zone of periventricular hypothalamus, Hv; and (**M**–**O**) the locus coeruleus, LC. The left column depicts the glucocorticoid GRα expression in green, the middle column depicts the expression of the β_2_-AR ir with red, the third column depicts the co-localization of β_2_-ARs with GRα, shown more precisely in inserts of higher magnification. Scale bar = 0.05 mm.

GRα-ir co-localization in β_2_-AR+ cells (Figure 5) was evident in the medial zone of the dorsal telencephalic area (Dm), the anterior (PPa) and posterior (PPp), part of the parvocellular preoptic nucleus, the ventral zone of periventricular hypothalamus (Hv), and importantly within the locus coeruleus (LC). Zebrafish locus coeruleus, a noradrenergic center, is suggested to be homologue to the mammalian locus coeruleus, A6 group [52], and was previously shown to express β_2_-ARs [41].

**Figure 6:**
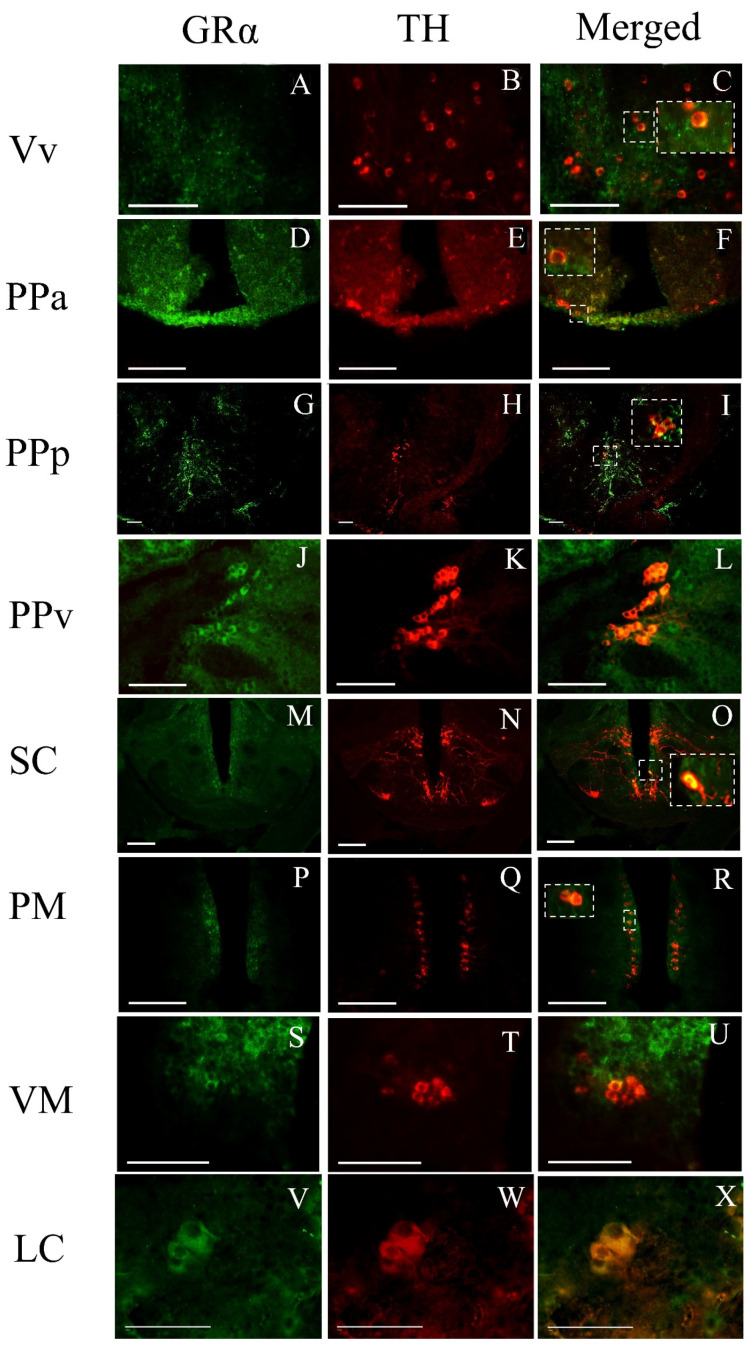
Double immunofluorescence of cells expressing GRα-ir co-localized with the TH in zebrafish brains. Double-labeled cells were found in (**A**–**C**) the ventral nucleus of ventral telencephalic area, Vv; (**D**–**F**) the anterior part of parvocellular preoptic nucleus, PPa; (**G**–**I**) the posterior part of parvocellular preoptic nucleus; (**J**–**L**) the ventral part of periventricular pretectal nucleus, PPv; (**M**–**O**) the suprachiasmatic nucleus, SC; (**P**–**R**) the magnocellular preoptic nucleus PM; (**S**–**U**) the ventromedial thalamic nucleus, VM; and (**V**–**X**) the locus coeruleus (LC). The left column depicts the glucocorticoid GRα expression in green, the middle column depicts the expression of the TH protein with red, the third column depicts the co-localization of TH protein with GRα, shown more precisely in inserts of higher magnification. Scale bar = 0.05 mm.

In addition, GRα is co-localized with TH (Figure 6), labelling dopaminergic cells [53,54]. Specifically, TH-expressing cells were double-labeled with GRα in the ventral nucleus of ventral telencephalic area (Vv), the preoptic areas (PPa and PPp), the ventral part of periventricular pretectal nucleus (PPv), the suprachiasmatic nucleus (SC), the magnocellular preoptic nucleus (PM), the ventromedial thalamic nucleus (VM) and the periventricular nucleus of the posterior tuberculum (TPp), the lateral hypothalamic nucleus (LH) and the noradrenergic nucleus, and the locus coeruleus (LC). TPp has characteristic large catecholaminergic cells, some of them known to project to the subpallium and are suggested to be homologues to a diencephalic division of the mammalian ascending mesodiencephalic dopaminergic groups A8–A10 [53].

**Figure 7:**
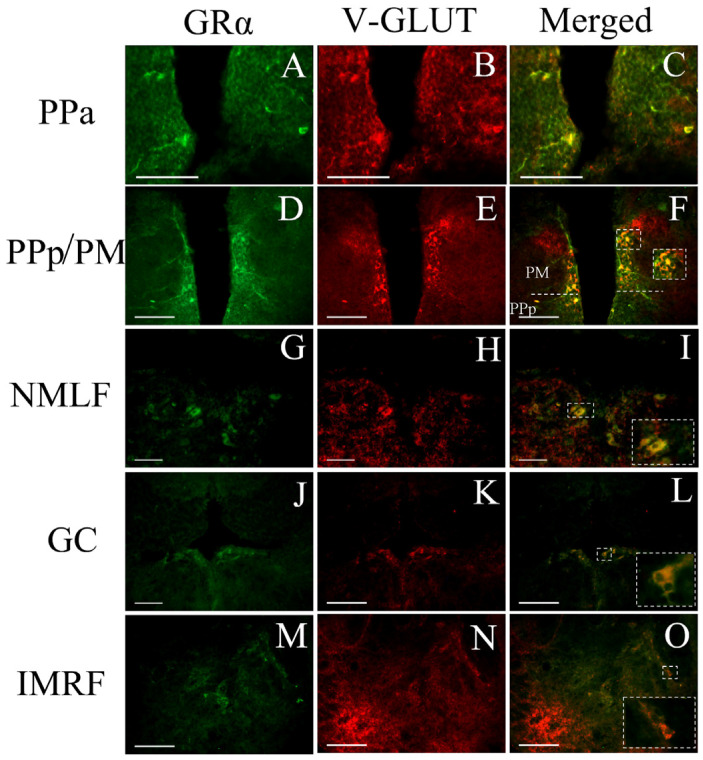
Double immunofluorescence of cells co-expressing GRα with the vGLUT2 protein in zebrafish brains. (**A**–**C**) Double labelling in the anterior parvocellular preoptic nucleus, PPa; (**D**–**F**) the posterior parvocellular, PPp, and magnocellular preoptic nucleus, PM; (**G**–**I**) the nucleus of medial longitudinal fascicle, NMLF; (**J**–**L**) the central gray, GC; and (**M**–**O**) the intermediate reticular formation, IMRF. The left column depicts GRα expression, the middle column depicts the expression of the V-GLUT2 expression with red, while the third column depicts the co-localization of vGLUT2 with the GRα, shown more precisely in inserts of higher magnification. Scale bar = 0.05 mm.

Moreover, GRα is co-localized with the vGLUT2 protein (Figure 7), expressed in glutamatergic cells [55]. Double immunofluorescence experiments demonstrated that GRα is co-expressed with vGLUT2 in cells of the periventricular gray zone of optic tectum, of both the anterior (PPa) and posterior (PPp) parvocellular preoptic nucleus, of the magnocellular preoptic nucleus (PM), of the central gray (GC), of the superior reticular formation (SRF), of the nucleus of medial longitudinal fascicle (NMLF), and of the intermediate reticular formation (IMRF) cells. Interestingly, the large GRα-immunoreactive cells in the brainstem nuclei SRF, IMRF, and IRF, were also v-GLUT-positive (glutamatergic cells).

**Figure 8:**
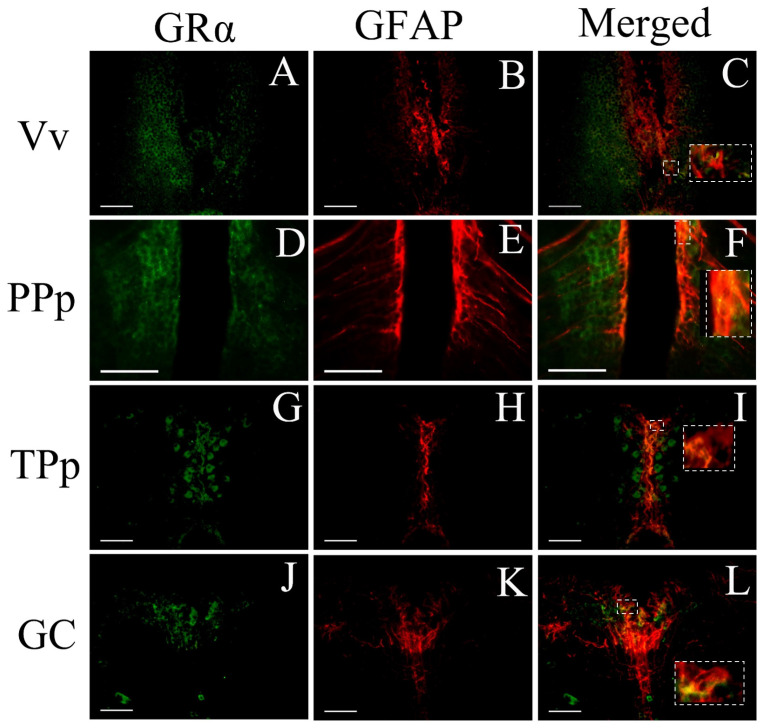
Double immunofluorescence of cells co-expressing GRα with the GFAP protein in zebrafish brains. (**A**–**C**) Co-localization in the ventral nucleus of ventral telencephalic area, Vv; (**D**–**F**) posterior parvocellular preoptic nucleus, PPp; (**G**–**I**) the periventricular nucleus of posterior tuberculum, TPp; and (**J**–**L**) the central gray, GC. The left column depicts GRα expression, the middle column depicts the expression of the glial GFAP in red, while the third column depicts the co-localization of GFAP with GRα, shown more precisely in inserts of higher magnification. Scale bar = 0.05 mm.

Importantly, in some cases, GRα receptors were found to be expressed in GFAP-positive cells (Figure 8). Glial fibrillary acidic protein, GFAP, is an astrocyte-specific member of the family of intermediate filament proteins taking place in formation of cytoskeletal structure, indicating the glial nature of GFAP-expressing cells [56]. The GFAP gene in zebrafish has the same exon–intron organization as the mammalian orthologue genes [57]. Double-labeling immunofluorescence experiments demonstrated the partial co-localization of GRα and GFAP in the periventricular zone of ventral area of ventral telencephalon (Figure 8A–C). A percentage of GRα-positive cells expressed GFAP in the medial zone of the dorsal telencephalic area (Dm), the lateral zone of the dorsal telencephalic area (Dl), and ventral zone of periventricular hypothalamus (Hv). In addition, GRa positive cells were in close proximity with neighboring GFAP positive fibers in PPp (Figure 8D–F). Double labeling was also observed in the periventricular nucleus of the posterior tuberculum that also exhibited GRα-ir adjacent to neighboring GFAP+ fibers (TPp; Figure 8G–I) and in the central gray area (GC; Figure 8J–L). Moreover, the periventricular layer of optic tectum (SPV) exhibited both GRα and GFAP immunoreactivity, but due to the high intensity of GFAP immunofluorescence, we could not determine double labeling.

**Figure 9:**
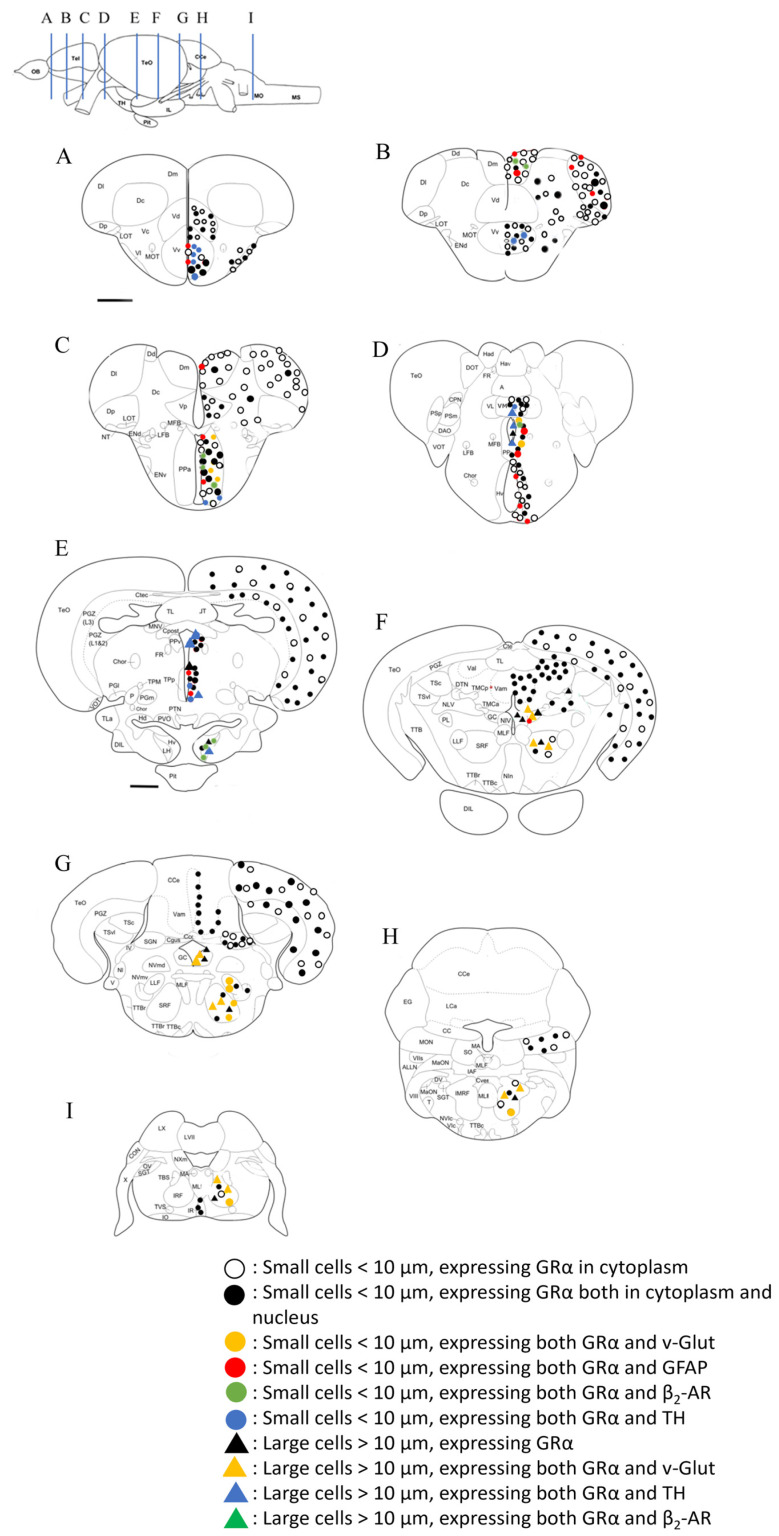
Schematic illustration of the GRα immunoreactivity distribution pattern and double labeling at indicative anterior to posterior coronal levels (**A**–**I**), shown in upper left image of the atlas of Wullimann et al., 1996 [47]. Labels are summarized in the left lower end. Every symbol represents 1–5 GRα positive cells.

A summary of the distribution and phenotype of GRα-expressing cells in selected coronal sections of zebrafish brains, using the atlas of Wullimann et al., 1996 [47], across the antero-posterior axis, is illustrated in Figure 9. 

## 4. Discussion

The present study revealed the existence of four main distinct immunoreactive protein bands, 95, 60, 45 and 35 kDa, of the glucocorticoid receptor GRα in zebrafish brains, with the band at approximately 95 kDa showing higher immunoreactivity in the forebrain compared to the midbrain and hindbrain. Cortisol or synthetic glucocorticoids activate zebrafish GRα, mediating gene transcription similarly to human GRα [22,26,58]. In zebrafish, GR is encoded from a single gene, highly similar to the organization of the human gene, and many GR proteins are produced due to the alternative splicing process and the alternative translation start position [22,25,58,59]. Particularly, recent evidence suggests that glucocorticoid signaling mediates long lasting effects of early life stress in zebrafish, as is the case in mammals [60]. However, using zebrafish embryos, MO knockdown of the GRα, revealed a differential potential to regulate target genes depending on the condition; that is, under basal activity, regulated genes involved in cell cycle and apoptosis while under stress condition, increased activation of GRα-regulated metabolic genes [59]. Interestingly, transcriptomic studies in early development using GR knockdown showed that GR signaling had major impact on zebrafish morphogenesis, including brain developmental events, such as telencephalic and hypothalamic neurogenesis, and patterning [61,62]. Importantly, a study using adult viable zebrafish mutant lacking all GR genomic activity suggests the evolutionary conserved role of glucocorticoid signaling in emotional disorders [26]. Whether the immunoreactive proteins detected here in zebrafish brains have similar functions or differentially regulate the regional-specific GR responses to glucocorticoids remains to be determined.

In addition, the distribution of GRα immunoreactivity in the zebrafish telencephalic, preoptic, hypothalamic, and brainstem areas showed significant similarities to previous reports in other teleost fishes [31,32], as well as in mammals [30,63,64,65], in frogs [66], and in Japanese quail [67]. Moreover, the present study identified populations of β_2_-AR+, TH+, vGLUT2+ and GFAP+ cells expressing GRα, indicating a possible modulation of dopaminergic and/or noradrenergic and glutamatergic transmission by glucocorticoids in zebrafish brains. 

### 4.1. GRα-Immunoreactive Proteins in Zebrafish Brain

Recent studies in human tissue demonstrated a functional role of the different glucocorticoid receptor isoforms GRα-A, GRα-B, GRα-C1, GRα-C2, GRα-C3, GRα-D1, GRα-D2, and GRα-D3 [5,12]. GRα translational isoforms show similar affinity for glucocorticoids and a similar ability to interact with GREs response elements following binding to ligands [5,10]. GRα-A, GRα-B, and GRα-C isoforms are located in cytoplasm of the cells, in the absence of the hormones and are shifted to the nucleus after binding to glucocorticoids. In contrast, GRα-D isoforms are permanently present in the cell nucleus and do not have the entire AF1 structure (a strong transcriptional activation region, which is important for maximal transcriptional enhancement), therefore they have a reduced ability to induce transcription and remain in the nucleus independent of the binding of a ligand [10,68]. 

An immunoreactive band at 95 kDa, considered isoform 1, was detected, with significantly higher expression in the forebrain, possibly representing the full-length isoform in zebrafish brain. Similarly, the immunoreactive 95 kDa band was identified as an GRα isoform in many studies [5,8,10,40,69,70,71]. In support of this, a 90–95 kDa GRα-immunoreactive band was detected in a zebrafish larvae/embryo [37,38,72]. GRα full-length immunoreactivity at 95–100 kDa, is suggested to correspond to the GRα-A identified previously in mice [5]. In agreement, the majority of GRα expressing cells in the forebrain areas showed a cytoplasmic localization of the immunohistochemical labeling. In human cortex, the 97 kDa isoform shows an age-related downregulation, suggested to act as a possible mechanism for resistance to glucocorticoids [33]. The function of this full-length immunoreactive protein in the adult zebrafish forebrain is not yet well known, but it may be related to stress plasticity and lifelong developmental mechanisms, e.g., evidence supports that it is controlling the epithelial calcium channel and is downregulated in GR morpholino oligonucleotide knockdown zebrafish embryos [37]. Anxiety following early life stress is dependent on glucocorticoid signaling in zebrafish.

### 4.2. GRα Immunoreactivity in Stress-Related Brain Areas

Expression of the GRα receptor showed a wide distribution in distinct groups of cells in zebrafish brains. Large-sized labeled cells showed GRα expression mostly in the cytoplasm, while a percentage of medium- and small-sized cells showed both cytoplasmic and nuclear labeling. Interestingly, a high percentage of GRα-positive cells characterized key areas controlling stress responses, including the amygdala, hippocampus, preoptic area and hypothalamus. Specifically, the amygdala activates the HPA axis [73], inducing hypercortisolemia [74,75], while the hippocampus inhibits the stress axis [76,77].

The zebrafish medial zone of dorsal telencephalic area (Dm), suggested to correspond to mammalian basal amygdala, was found to include high number of GRα-immunoreactive cells, in agreement to studies of glucocorticoid receptors’ expression in the homologue structure of the salmon telencephalon [31]. In support, GR expression has been reported in rat amygdala [30], while high levels of GR mRNA were shown in the amygdala of squirrel monkey brains [27]. Zebrafish Dm is considered part of the mesolimbic reward circuitry involved in emotional memory processes and induction of motivated behavior [49,78,79,80,81] and has been shown to exhibit sex-specific dimorphic neurogenetic potential [82]. Importantly, a disruption of GR causes a syndrome in adult zebrafish that resembles an affective disorder, with the molecular signature of chronic stress and a behavioral profile of depression [26], possibly involving Dm GRα+/β2-AR expressing cells. In addition to neuronal expression, GRα immunoreactivity in zebrafish Dm astroglial cells may have a role in emotional behavior. In support, GR-containing astrocytes in human amygdalae are increased in postmortem studies in major depression [83]. 

In addition, GRα immunoreactivity characterized zebrafish lateral zone of dorsal telencephalic area (Dl), homologue to the mammalian hippocampus [78,79], which suggests it to be involved in spatial learning and short-term memory procedures [27,84,85]. In agreement, Carruth and her colleagues (2000) [31] demonstrated the expression of glucocorticoid receptors in ventral-lateral and lateral parts of the dorsal telencephalon of salmon. GR expression in mammalian hippocampus has been shown in adult rhesus monkeys [28] and in rats [30], as well as high levels of GR mRNA (of the full-length alpha isoform of GR protein) in CA1 and CA2 of squirrel monkey hippocampi [27]. Mammalian hippocampal function is significantly influenced by the concentration of glucocorticoids. Acute administration of glucocorticoids regulates neuronal excitability and alters glucocorticoid-dependent behaviors, while chronic glucocorticoid administration affects hippocampal morphology leading to cognitive impairment by activation of MR and GR receptors, inhibiting neuronal excitability [86]. Moreover, glucocorticoids possibly influence adult neurogenesis in the dentate gyrus of mammalian hippocampus [87] as well as, in the dorso-lateral telencephalon of teleost fish [88,89], further suggesting their conserved features in vertebrate hippocampus. 

Significant GRα immunoreactivity was also detected in large-sized, heavily stained cells of the preoptic, posterior tuberculum and hypothalamic areas of zebrafish, suggesting that glucocorticoid receptors in hypothalamic-key areas influence a wide range of brain functions. In agreement, previous studies have shown the GRα expression in rats [29,30], in adult rhesus monkeys (Macaca mulatta) [28], as well as the hypothalami of salmon [31]. Most of these areas are characterized as dopaminergic neuromodulatory centers based on the TH expression [54], with the preoptic region and the posterior tuberculum strongly expressing both TH genes [42]. Indeed, the double labeling of GRα and TH characterized zebrafish anterior preoptic area, lateral hypothalamus, and posterior tuberculum. While a complex dopaminergic phenotype has been proposed, based on differential expression pattern of TH1 and TH2 genes, dopamine transporter and vesicular monoamine transporter 2 [42], the present study cannot differentiate the expression of GRα in the different dopaminergic phenotypes. Whether there is a differential influence of glucocorticoids in these dual transmitter dopaminergic phenotypes remains to be determined. Moreover, GRα-ir in the posterior part of parvocellular preoptic nucleus (PPp), the periventricular nucleus of posterior tuberculum (TPp), and the ventral zone of periventricular hypothalamus is closely associated with radial glial fibers, possibly influencing the neurogenetic potential of these areas.

An interaction of the glucocorticoid receptors and noradrenergic transmission, possibly contributing to allostatic stress mechanisms, is supported by the GRα-ir in β_2_-AR-expressing cells in the locus coeruleus neurons. Locus coeruleus neurons supply most of the noradrenergic input to the brain areas [52], suggesting the modulation of their activity by glucocorticoids. In addition, preoptic the areas and periventricular hypothalamus include GRα+/β2AR+ cells, while most of the GRα-ir zebrafish brain areas exhibit moderate to high expression of both α2-Ars [43] and β2-Ars [41]. An interplay of noradrenergic and hormonal stress responses has been suggested to contribute to stress plasticity mechanisms underlying the long-term effects of early life stress on seabream Dm amygdalae [90]. In support, the mammalian locus coeruleus (LC) includes a high density of GR-immunoreactive cells [29,30].

### 4.3. GRα Expression in Social Behavior/Reward Brain Network

The neural substrates regulating social behavior, described as the “social behavior network” (SBN), is suggested to be evolutionary conserved across vertebrates [91]. Most of the key areas of the SBN are also part of the mesolimbic reward system. These areas, known to be involved in the control of multiple forms of social behavior (e.g., reproductive behavior, aggression), include the lateral septum, preoptic area, ventromedial hypothalamus (VMH), and the central gray (CG). Interestingly, our study showed a significant expression of glucocorticoid receptors in most of the identified SBN areas. 

In addition to the GRα expression in the preoptic and hypothalamic areas (discussed above), glial cells were found to express GRα within the ventral telencephalic region (Vv) a key-area implicated in social behavior [92] and the reward mesolimbic system, proposed to correspond to the mammalian lateral septum [49,91]. In support, Vv includes high density of β_2_-ARs [41] and high α_2A_-AR levels [43], and is involved in sex-specific swimming behavior [93]. Furthermore, the rhombencephalic central gray (CG), suggested to be involved in several essential physiological processes, including reproductive behavior, visceral animal responses, and analgesia, exhibited significant expression of GRα. Importantly, the central gray contains high densities of β_2_-AR-immunoreactive cell somata and fibers in adult zebrafish and red porgy brain [93,94]. 

### 4.4. GRα Immunoreactivity in the Cerebellum

The somata of Purkinje cells that integrate mossy and climbing fibers signals, show dense immunoreactivity for GRα. This labeling pattern in cerebellar circuitry, indicates that GRα have the potential to regulate cerebellum motor learning, coordination and multisensory integration in zebrafish. Indeed, the teleostean cerebellum, has a role in spatial and emotional learning [95], motor coordination and sex-specific swimming behavior [93]. GRα immunoreactivity was also evident in the rhombencephalic medial octavolateralis nucleus (MON), which conveys sensory information [96] (Bell, 1981) to cerebellar granule cells via mossy fibers [97]. GRs have been shown to be expressed in the mammalian cerebelli, rat [29,30], and rhesus macaques [28], while high levels of GR mRNA were detected in the cerebellum of squirrel monkey [27]. The GR expression pattern in teleost fish Pagrus major [98] and in zebrafish (the present study) is further supporting the evolutionary conserved role of glucocorticoid receptors in cerebellar function. 

### 4.5. GRα Expression in Astroglial Cells

Recent evidence suggests that astrocytic GRs play an important role in stress responses, with reduced astrocytic GR expression to associate to stress vulnerability, while restoring astrocytic GR expression in the medial prefrontal cortex prevents depressive-like phenotype [99]. The GFAP-positive cells, astroglial cells, in the fish brain displaying morphological characteristics of radial glia [100] show similar functions with those reported for mammalian glial cells, e.g., during regeneration, synaptic plasticity, neurogenesis and reactive gliosis in health and disease [101,102]. In zebrafish, GRα immunoreactivity close to the midline was associated with GFAP positive radial glial cells in the ventral nucleus of ventral telencephalic area (Vv), the posterior part of parvocellular preoptic nucleus (PPp), the periventricular nucleus of posterior tuberculum (TPp), the ventral zone of periventricular hypothalamus and the central gray (GC). In addition, the dense radial glial processes of the stratum periventriculare (SPV) of the optic tectum [103] were in close association to GRα immunoreactivity. While the majority of GFAP expressing glial cells/astroglia exhibit morphological characteristics of mammalian radial glia in ventricular neurogenetic zones of adult zebrafish brain, star-shaped cells and radial extensions have also been reported to be somewhat similar to mammalian astrocytes [104]. Although the specific phenotype (astrocytes or radial glia) of GRα-ir glial cells has not been precisely determined, recent evidence in zebrafish supports that acute stress may activate A1 astrocytes, which can exert adverse effects on neural circuits, as A1 cells lose normal astrocyte functions (e.g., enhancing neuronal survival) and release neurotoxic factors [105]. Interestingly, the midline astroglia within the PPa, PPp and TPp nuclei is characterized by high levels of adrenergic receptors [41,43], possibly representing a potential site for interaction of adrenergic and glucocorticoid receptors, modulating brain homeostasis during coping to environmental challenges. This evidence complements previous data from mammals [106] and highlights the possible role of glial cells as a cellular target of therapies of stress-induced brain diseases.

## 5. Conclusions

The present study suggests that the wide distribution pattern of GR expression in various brain structures in the zebrafish brain is comparable to other vertebrates. Specifically, GRα immunoreactivity is evident in various brain regions that are known to be involved in stress plasticity, social behavior, and integration of sensory and motor information. In addition, the co-localization of GRα with catecholaminergic and glutamatergic neurons further supports the evolutionally conserved features of glucocorticoid receptors in zebrafish brains and suggests their potential to modulate the specific neurotransmitter functions in key brain structures. Moreover, the GRα expression in astroglia/radial glia, suggests an additional functional site for glucocorticoids in maintaining brain homeostasis. Given the high conservation of GRα between zebrafish and humans, these findings expand our knowledge on brain glucocorticoid receptors and complement mammalian models in translation research of stress-related disorders.

## Data Availability

The data presented in this study are available upon request from the corresponding author.

## Supplementary Materials

The following supporting information can be downloaded at: https://www.mdpi.com/article/10.3390/brainsci13060861/s1, Figure S1: Western immunoblotting showing the quantitative expression of glucocorticoid receptor (GRα) isoforms in mammalian and teleostean brains; Table S1: Details of the primary antibodies used.
