# Supplementary material for: Neuronal and Astroglial Localization of Glucocorticoid Receptor GRα in Adult Zebrafish Brain (Danio rerio)
Source: Brain Sci. 2023 May 26;13(6):861. doi: 10.3390/brainsci13060861 (PMC10296162; doi:10.3390/brainsci13060861)
Supplement: Supplementary file 1 [file brainsci-13-00861-s001.zip › brainsci-2372581-supplementary.pdf]

Supplementary Table S1. Antibodies and their concentrations used.

Antibody	Source	Host species	Dilution
GRα rabbit polyclonal antibody	**(Santa Cruz Biotechnology, Cat# sc-1002, RRID: AB_2155788)**	Rabbit	1:100 in PBS with 0.5% Triton X-100
α- tubulin antibody	**(Sigma-Aldrich Cat# T5168, RRID: AB_477579)**	Mouse	1:6000 in TBS-Tween with 2% nonfat dried milk
monoclonal anti-TH antibody	**(Chemicon, Cat# MAB318, RRID: AB_2201528)**	Mouse	1:1000 in PBS with 0.5% Triton X-100
monoclonal anti-GFAP antibody	**(Sigma, clone G-A-5 Cat# G3893, RRID: AB_477010)**	Mouse	1:1000 in PBS with 0.5% Triton X-100
polyclonal anti- v-GLUT antibody	**(Millipore, Cat# MAB5504, RRID: AB_2187552)**	Mouse	1:1000 in PBS with 0.5% Triton X-100
monoclonal antibody against anti-β2-AR	**(Santa Cruz, Cat# sc-81577, RR11E1, RRID:AB_1119478)**	Mouse	1:300 in PBS with 0.5% Triton X-100

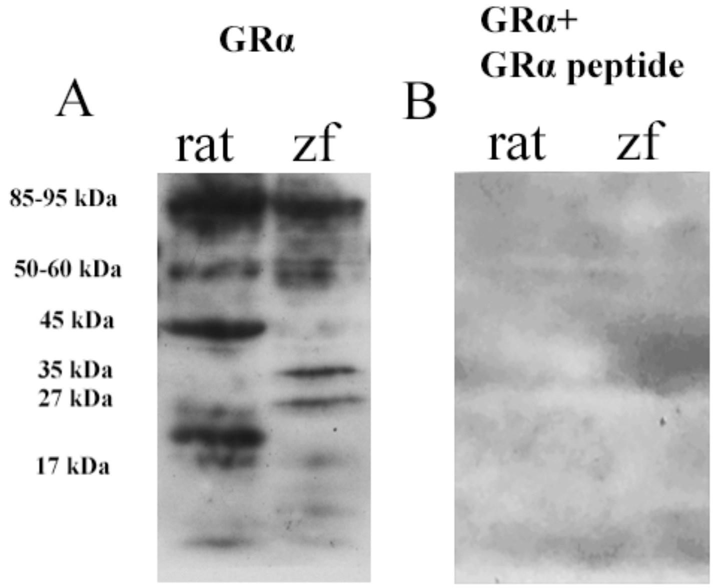

Supplementary Figure S1. Western immunoblot experiments to compare the migration of the GRα immunoreacting proteins in mammalian and teleostean brains.
